# Evaluating the effectiveness of structural and metabolic tooth enamel reparation by magnesium-calcium remineralizing complex

**DOI:** 10.1186/1878-5085-5-S1-A122

**Published:** 2014-02-11

**Authors:** Anatoly A Kunin, Irina A Belenova, Tatyana V Kupets

**Affiliations:** 1 Professors, Dr. Med. Sc., Voronezh N.N. Burdenko State Medical Academy, Therapeutic Dentistry Department, Voronezh, Russia; 2 DRC-group, Moscow, Russian-Switzerland comany, Russia

## 

The prevention and treatment of caries and non-carious lesions by means of remineralization has been studied for many decades. The theoretical basis for remineralization as a means of caries prevention and treatment is based on the retention of protein matrix in dental enamel at early stage of caries (white spot) and on the possibility of its remineralization.

The detection of new structural formations and emerging information about morph-chemical properties of human and animal enamel have facilitated identification of a new approach not only to for caries prevention but also the creation of new, more effective formulations for oral health hygiene as well as, structural and metabolic reparation of tooth enamel.

For ultrastructure and morph-chemical tooth enamel reconstruction, we applied dentifrice gel ROCS Medical Minerals which contains calcium glycerophosphate, magnesium chloride and xylitol. Participants were 10–30 years old, without any significant medical conditions. Each patient received 15 applications of the gel. Total of 57 subjects were divided to 4 groups depending on the diagnosis. All the subjects had indications for remineralizing therapy.

• The 1^st^ group consisted of 15 patients with early stage of caries (white spot).

• The 2^nd^ group was made up of 19 patients with systemic hypoplasia (spotted form).

• The 3^rd^ group consisted of 10 young subjects with significant hypersensitivity without any visible lesions (the functional insufficiency of enamel, according to IG Lukomsky).

• The 4^th^ group consisted of 13 subjects with hypersensitivity due to wedge-shaped lesions, abrasion or enamel erosion.

## Results and discussion

The application of gel containing the new mineral complex of magnesium and calcium resulted in a positive remineralizing effect which was obtained in patients with early stages of caries as well as those with non-caries lesions. Further, white spots (early stage of caries) completely disappeared in 80% cases after 15 applications of the dentifrice. Thus, it appears that 15 procedures of remineralizing therapy with ROCS Medical Minerals are enough for mineralizing of white hypoplastic spots. Most patients with hypersensitivity (82% and 93% in two groups) noticed significant reduction of painful reactions after two 15-minutes applications. Finally, all subjects noticed a significant improvement of teeth appearance (i.e., whitening and brilliance enhancement).

In conclusion, the results suggest high efficacy of the mineral magnesium-calcium remineralizing complex in patients with early stages of caries as well as those with non-carious lesions.

